# The SLMTA programme: Transforming the laboratory landscape in developing countries

**DOI:** 10.4102/ajlm.v3i2.194

**Published:** 2014-09-16

**Authors:** Katy Yao, Talkmore Maruta, Elizabeth T. Luman, John N. Nkengasong

**Affiliations:** 1 International Laboratory Branch, Division of Global HIV/AIDS, US Centers for Disease Control and Prevention (CDC), Atlanta, United States; 2 African Society for Laboratory Medicine (ASLM), Addis Ababa, Ethiopia

## Abstract

**Background:**

Efficient and reliable laboratory services are essential to effective and well-functioning health systems. Laboratory managers play a critical role in ensuring the quality and timeliness of these services. However, few laboratory management programmes focus on the competencies required for the daily operations of a laboratory in resource-limited settings. This report provides a detailed description of an innovative laboratory management training tool called Strengthening Laboratory Management Toward Accreditation (SLMTA) and highlights some challenges, achievements and lessons learned during the first five years of implementation (2009–2013) in developing countries.

**Programme:**

SLMTA is a competency-based programme that uses a series of short courses and work-based learning projects to effect immediate and measurable laboratory improvement, while empowering laboratory managers to implement practical quality management systems to ensure better patient care. A SLMTA training programme spans from 12 to 18 months; after each workshop, participants implement improvement projects supported by regular supervisory visits or on-site mentoring. In order to assess strengths, weaknesses and progress made by the laboratory, audits are conducted using the World Health Organization’s Regional Office for Africa (WHO AFRO) Stepwise Laboratory Quality Improvement Process Towards Accreditation (SLIPTA) checklist, which is based on International Organization for Standardization (ISO) 15189 requirements. These internal audits are conducted at the beginning and end of the SLMTA training programme.

**Conclusion:**

Within five years, SLMTA had been implemented in 617 laboratories in 47 countries, transforming the laboratory landscape in developing countries. To our knowledge, SLMTA is the first programme that makes an explicit connection between the performance of specific management behaviours and routines and ISO 15189 requirements. Because of this close relationship, SLMTA is uniquely positioned to help laboratories seek accreditation to ISO 15189.

## Introduction

Efficient and reliable laboratory services are essential to a functioning health system as high-quality laboratory testing plays a key role in patient care, surveillance and outbreak investigation.^1^ Poor laboratory quality and its negative impact on healthcare systems have been documented for resource-limited settings, including sub-Saharan Africa (SSA).^2,3,4,5^ Using the number of accredited laboratories as a quality metric, a 2013 survey showed that 37 out of the 49 countries in SSA had no medical laboratories accredited to any internationally-recognised standards. Of the 380 accredited laboratories in that region, 91% were in South Africa and only 17% were public health laboratories.^6^

In recent years, however, several landmark events have drawn attention to the poor state of public health laboratories and have pushed for strengthening of laboratory systems and networks.^1,7^ One of these events was the issuance of the World Health Organization (WHO)–Lyon statement in 2008,^8^ which called for countries with limited resources to pursue practical quality management systems and to adopt a stepwise approach to quality improvement and accreditation.^4,7^ Another was the 2009 launch of a laboratory management training programme called ‘Strengthening Laboratory Management Toward Accreditation’ (SLMTA).^1^

Effective management and leadership are critical to strengthening health systems and the scaling up of health service delivery.^9^ Recently, many countries and partners have initiated efforts to enhance management of health programmes and service delivery in developing countries, with measurable success.^10,11,12,13,14,15,16,17,18^ Most of these management capacity-building efforts focused on managers from hospitals, primary healthcare centers (such as family planning, mother–child health, etc.), or vertical public health programmes (such as tuberculosis [TB] and HIV). Existing laboratory management capacity-building efforts have primarily targeted senior laboratory officials where the focus is on laboratory policy, system and network development,^19,20,21,22,23^ as opposed to daily operations of individual laboratories. Training programmes are needed to enable laboratory managers to use available resources (staff, budgets, supplies, equipment, buildings and information) efficiently for planning, implementation and evaluation of service delivery in order to meet patients’ and clinicians’ expectations and public health needs.^24^

The SLMTA programme was created in response to the observed need for structured laboratory management training and quality improvement by the US Centers for Disease Control and Prevention (CDC), in collaboration with the American Society for Clinical Pathology, the Clinton Health Access Initiative, and the World Health Organization’s Regional Office for Africa (WHO AFRO). SLMTA is a competency-based management training programme which uses a series of short didactic courses and work-based applied learning projects with the goal of achieving immediate and measurable laboratory improvements. It provides a practical approach to addressing everyday challenges using available resources.

The SLMTA training curriculum and implementation method were pilot-tested in 15 laboratories in Uganda from August 2008 to March 2009, yielding promising results.^24^ SLMTA was then officially launched in 2009, with implementation beginning in 2010. As of the end of 2013, SLMTA had been rolled out in 47 countries and 617 laboratories, and had improved enrolled laboratories an average of 23 percentage points after one round of SLMTA training in a pre/post study using the WHO AFRO Stepwise Laboratory Quality Improvement Process Towards Accreditation (SLIPTA) checklist.^25^ This report provides a detailed description of the SLMTA programme and highlights some challenges, achievements and lessons learned during its first five years of implementation (2009–2013) in developing countries.

## Key components

**FIGURE 1:**
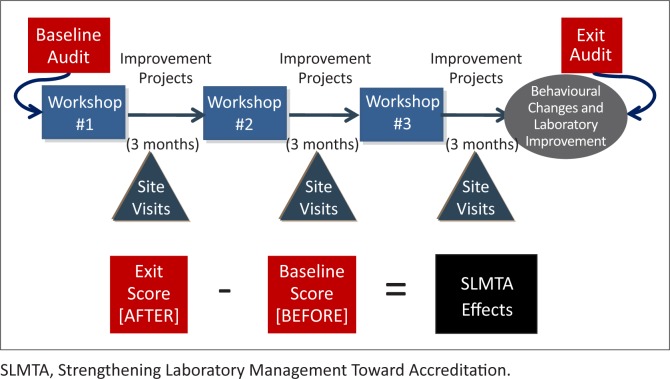
Standard SLMTA implementation process.

The design of the SLMTA curriculum and its implementation exemplify what is known as ‘good practice’ in management competencies development.^19,26^ The SLMTA curriculum covers the 10 key competencies of a laboratory manager: productivity; work area; inventory; procurement; equipment maintenance; quality assurance; specimens; laboratory testing; test result reporting; and document and records control. A total of 66 tasks and job routines define effective laboratory management and constitute the learning objectives of the curriculum.^24^ A typical SLMTA training programme spans from 12 to 18 months (Figure 1). Training is conducted in a series of three workshops, each lasting three to four days, utilising 44 instructional activities^27^ and more than 100 job aids. Each activity provides hands-on, practice-based learning experience for specific management tasks. The total training time is approximately 60 hours to teach all 44 activities.

After each workshop, participants implement improvement projects in their home laboratories. There are two types of improvement projects: complicated projects that require extensive planning and data collection before and after the change; and simpler ‘just do it’ types of projects that can be implemented immediately with minimal time and resources (Box 1). Implementation of improvement projects requires teamwork involving the entire laboratory staff, thus ensuring that the projects become part of the laboratory’s continuous improvement processes. Participants are encouraged to implement locally-appropriate solutions using existing resources. During the home-based learning period after each workshop, participants are supported by periodic supervisory visits or on-site mentoring guided by standardised tools. This structured supervision and support component is critical to the success of the SLMTA programme.

BOX 1 Examples of improvement projects.

**Table:** 

**Simple ‘just do it’ projects** Apply the 6 S’s (Sort, Straighten, Shine, Standardise, Sustain and Safety) in an area of the laboratory (storeroom, a work station, etc.) Implement a duty roster Create a management calendar Create an equipment and reagent master list/inventory Conduct regular staff meetings
**Projects that require extensive planning and data collection before and after change** Monitor one of the quality indicators from the Balanced Scorecard activity. Redesign your floor plan to improve efficiency and measure the change such as reduction in turn-around time. Design a competency assessment programme and conduct a set number of assessments. Conduct a safety audit and reduce the number of identified non-conformities. Introduce an inventory management system; monitor stock-outs. Implement equipment maintenance and service. Improve documentation (policies, standard operating procedures, quality logs, checklists, etc.). Monitor running of internal quality control. Monitor performance and documentation of External Quality Assessment. Monitor and reduce specimen rejection rates. Monitor results of referral specimens. Conduct customer satisfaction survey and follow up on issues.

The formal laboratory evaluation component is designed to identify weaknesses and areas that require improvement, measure success of the programme and indicate future goals for the laboratory. Evaluations are based on WHO AFRO’s five-stage accreditation-preparedness scheme, called SLIPTA, which recognises laboratories according to their level of compliance with the International Organisation for Standardization (ISO) 15189 standard.^1^ Under the SLIPTA scheme, laboratories are audited using the SLIPTA checklist, which includes 111 items divided into 12 sections (Table 1) based on the 12 Quality System Essentials from the Clinical and Laboratory Standards Institute (CLSI).^28^ After an audit, a laboratory receives a score out of 258 points in order to determine its star rating – from ‘0’ (0–141 points, < 55%) to ‘5’ (244–258 points, ≥ 95%).^29^ Not all laboratories will pursue accreditation; regardless, the SLIPTA scheme provides the roadmap and motivation for laboratories to make steady improvement in service delivery and patient care.

**TABLE 1:** Sections of the WHO AFRO SLIPTA checklist and star ratings.

Section	Points
1. Documents and records	25
2. Management reviews	17
3. Organisation and personnel	20
4. Client management and customer service	8
5. Equipment	30
6. Internal audit	10
7. Purchasing and inventory	30
8. Process control and internal and/or external quality assessment	33
9. Information management	18
10.Corrective action	12
11.Occurrence and/or incident management and process improvement	12
12.Facilities and safety	43
**Total score**	**258**

Note: Star Rating; 0 Stars: 0–141 points, < 55%; 1 Star: 142–166 points, 55% – 64%; 2 Stars: 167–192 points, 65% – 74%; 3 Stars: 193–218 points, 75% – 84%; 4 Stars: 219–243 points, 85% – 94%; 5 Stars: 244–258 points, ≥ 95%.

WHO AFRO, World Health Organization’s Regional Office for Africa; SLIPTA, Stepwise Laboratory Quality Improvement Process Towards Accreditation.

SLMTA and SLIPTA are closely linked. The SLIPTA checklist provides the SLMTA programme with a means to identify gaps and benchmark progress. SLMTA, on the other hand, equips laboratory management with the ability to implement quality management systems in order to improve their performance on the SLIPTA scale and eventually achieve formal accreditation status. To support this link, individual SLIPTA checklist items are mapped to each of the 44 instructional activities in the SLMTA curriculum so that participants know exactly which management action will fulfill the requirements of any given checklist item. Because of this close linkage between the SLMTA curriculum and the SLIPTA checklist, in June 2012, after modification of the SLIPTA checklist, the SLMTA curriculum underwent revisions to remap the revised checklist items to SLMTA instructional activities.

Each laboratory participating in SLMTA conducts an internal audit at the beginning (baseline) and the end (exit) of the programme using the SLIPTA checklist. The difference between baseline and exit scores, as well as their respective star ratings, is calculated in order to quantify the effects of the programme on laboratory function and quality (Figure 1). In addition to the SLIPTA scores, laboratories demonstrate their progress through improvement project data such as turn-around time, sample rejection rate, stock out rate, customer satisfaction survey results and before-and-after photographs of physical changes.

## Variations from the basic implementation model

Some countries have customised SLMTA delivery to fit their local context. Two notable variations are Cameroon and Lesotho, which adapted their programmes to address local challenges and to enhance existing laboratory capacity-building efforts. Despite the variations, both adaptations adhere to the critical requirement of implementing SLMTA as a process (a series of workshops with improvement projects and mentoring) rather than a single training event.

### Cameroon

Most countries conduct the SLMTA training in a central location. This centralised model provides logistical convenience, particularly when many laboratories are enrolled in the same round, allowing the programme to train many laboratories at one time. It also enables personnel from various laboratories to interact and learn from each other. However, there are drawbacks, including, (1) high costs associated with renting a venue and travelling participants; (2) staff must be absent from their laboratories for prolonged periods because of travel between home and training locations; and (3) a limited number of staff can attend the course, creating a potential divide between those who are trained and those who are not. Working with a very limited budget, Cameroon decentralised the workshops and conducted facility-based training, with teams traveling to the laboratories in the programme to provide training on site. Whilst this model required more time from the trainers, it enabled hospital management and clinicians to be involved in the training alongside laboratory management, facilitating advocacy. In addition, it allowed the course to be better tailored to the needs of the individual laboratories, with all discussions related to site-specific challenges and solutions.^30^

### Lesotho

The schedule and frequency of trainings for the initial SLMTA round in Lesotho were modified in order to match existing mentorship timetables.^31,32^ At the time that SLMTA was adopted, the country had already begun a structured mentorship programme with an embedded mentor. This mentor soon became certified as a SLMTA trainer so that he could enhance on-going mentoring efforts with the SLMTA programme. These laboratories received SLMTA training one day per week over two blocks of six weeks each, spaced six months apart. The total training time was the same as the standard three-workshop model. Because of the availability of a full-time mentor, these laboratories received more intensive and frequent monitoring visits – a total of 12 visits versus the standard six – and were able to implement numerous improvement projects.

## Capacity building for programme scale-up

In order to facilitate programme scale-up, a training-of-trainers approach was used to develop indigenous trainers, who in turn implement the SLMTA programme in-country.^27^ Because the quality and integrity of the programme relies heavily on these local trainers, it is critical that they are competent and well qualified. To achieve that goal, the programme has established strict screening criteria in order to ensure that potential trainers have the necessary availability, motivation and commitment, along with a technical background. A formal training-of-trainers course was developed in which SLMTA master trainers teach both the curriculum content and also facilitation skills. This two-week course provides a demanding but supportive environment where participants conduct teach-back of assigned activities from the curriculum and immediately receive constructive feedback from master trainers in order to improve their facilitation skills and understanding of the content. To graduate, participants must fulfill several requirements: (1) 100% daily attendance, including group work sessions; (2) equal responsibility in the preparation and facilitation of teach-back assignments; (3) 100% completion of homework; and (4) endorsement by a master trainer. Participants and their organisations also receive reports providing performance reviews and recommendations on specific roles that they are competent to play in programme implementation.

Timely, specific, behaviour-focused feedback is the cornerstone of training-of-trainers. As such, the master trainers’ ability to mentor the participants and provide constructive feedback determines the quality of trainers produced. The rapid expansion of the SLMTA programme has resulted in the demand for more master trainers who can train trainers. Given the crucial role that master trainers play in developing competent trainers, they must be highly motivated and effective, their qualifications must be impeccable and their development and selection process rigorous. To be considered as a master trainer candidate, he or she must: (1) be a certified SLMTA trainer; (2) have conducted the entire SLMTA process; (3) have the availability and commitment needed to be a strong asset to the programme; and (4) be nominated by an existing master trainer. Eligible candidates are invited to a training-of-trainers course, where they apprentice under existing master trainers whilst sharing the course workload equally.^27^ Throughout the course, these candidates receive coaching and feedback on their performance from master trainers and their competence and commitment are assessed constantly.

## Additional considerations

### Country commitment

Countries adopting the SLMTA programme are advised to fulfill certain pre-requisites to ensure success. Firstly, they must have a national laboratory policy and strategic plan, along with a laboratory technical working group in order to drive the initiative forward. Secondly, countries must ensure financial and political support for SLMTA and a commitment to improving laboratory quality at all levels: Ministry of Health, hospital management, laboratory management and laboratory staff. It is critical that SLMTA sites have dedicated quality assurance and safety officers. It is also important for participants to remain in the same job or organisation throughout the duration of the programme and to be allowed the time needed to participate in the programme.

### Site selection

Site selection should be based on several factors, including facility infrastructure, staffing levels, impact on coverage of patient care, geographic considerations and demonstration of site commitment. The number of laboratories enrolled for each round of SLMTA (i.e., cohort) has varied by country – ranging from one each in Angola and Swaziland to 27 in Malawi.^25^ Countries have been advised to start small and scale up progressively. However, political pressure for broader impact and the desire for more laboratories to benefit from SLMTA may have resulted in some countries enrolling large numbers of laboratories. Four countries (Ethiopia, Malawi, Nigeria and Uganda) have enrolled > 20 laboratories in the first or subsequent SLMTA cohorts.^25^ Enrolling a large number of laboratories requires more human and logistical resources for the provision of sufficient site monitoring and support. In addition, it is essential that there is good communication and coordination amongst trainers and mentors so as to ensure consistency throughout the group.

Most countries have continued to enroll new laboratories in subsequent SLMTA cohorts.^25^ Kenya to date has initiated six cohorts of SLMTA, enrolling a total of 50 laboratories and seven blood banks. Lesotho, a small country with only 19 laboratories, has reached a high coverage of 18 (95%) laboratories over three cohorts of SLMTA.

### Human resources

Countries vary in their capacity to rollout the SLMTA programme. Implementation requires three primary cadres: trainers to teach the curriculum; auditors to perform the internal audits; and mentors to facilitate the improvement projects. Regional and in-country SLMTA training-of-trainer workshops conducted during the past five years have steadily produced more local trainers.^27^ Although the demand for SLMTA trainers still exceeds the supply, the deficiency is less severe than that of qualified auditors and mentors. Using unqualified auditors may lead to inaccurate audit findings and missed non-conformities. This gap is being addressed slowly as many countries are seeking partners’ help with regard to scaling up auditor training.

Mentorship and site visits may be the most challenging aspect of implementation and are often overlooked in the initial programme planning. Site visits require personnel time, transportation resources (fuel, vehicle, driver) and lodging and per diem if overnight stays are necessary. If this component is not scheduled and budgeted properly from the beginning, countries often struggle to provide the onsite support and supervision that are critical to the programme’s success. Site visits are necessary in order to check the progress of the improvement projects, assess effectiveness of the previous workshops, troubleshoot site-specific issues and provide motivation and encouragement. Site visits often involve meetings with top facility management to advocate support for the laboratory. The length of site visits has varied greatly between countries and even amongst laboratories within the same SLMTA cohort, ranging from half a day to three or more days at each site. The frequency and length of site visits should be considered carefully and planned according to the size and scope of testing activities in the laboratory. In addition, the level of quality at baseline and progress thereafter, as well as site staff’s experience with regard to implementing quality systems, should be considered. Laboratories needing more support should receive longer or more frequent visits to enable them to make measurable improvements and sustain their motivation.

The need for extensive but affordable site support has led countries such as Cameroon,^30^ Mozambique,^33^ Swaziland and Zimbabwe^34^ to establish structured mentorship programmes with full-time facility-based local mentors – a model spearheaded by Lesotho.^32,35^ This model has well-defined goals for each mentoring engagement, extended contact time on site, defined periods when mentors are absent, consistent approaches across laboratories and measurement of progress using standardised tools. Mentors may come from the laboratories they are assigned to mentor, from a local partner, or from outside the country. Mentors receive training in SLMTA implementation, mentorship and auditing. Because of their extended participation in the laboratories they are mentoring, they are able to gain knowledge of the rhythms, practices and personalities of the laboratory, enabling them to facilitate the necessary changes in attitudes and behaviours.

Other strategies have been used to provide the needed support for the SLMTA laboratories. In Kenya, for example, select SLMTA hospital laboratories were paired, or ‘twinned’, with internationally-accredited research laboratories. The accredited laboratories mentored the SLMTA laboratories in quality management system implementation.^36^

## Experience from Africa

SLMTA was launched in Africa in 2009. By the end of 2013, it had been implemented in 23 countries on the continent with a total of 503 participating laboratories, which constituted 87% of all the SLMTA-enrolled laboratories in the world.^25^ As the continent that launched SLMTA, Africa has demonstrated to the world that with ingenuity, innovation and determination, implementing quality management systems is possible, despite resource limitations. To date, four SLMTA-enrolled laboratories in Africa have been accredited to ISO 15189, whilst many more are making great progress in continuous quality improvement.^25^ In the sections below, we highlight the experiences of four African countries.

### Mozambique – Country ownership and sustainability

To develop a self-sufficient quality programme, Mozambique integrated SLMTA within the existing structure of the Ministry of Health laboratory system. A National Laboratory Quality Technical Working Group was established and a dedicated coordinator hired. The Ministry of Health provided the vision and leadership in implementation and advocacy, coordinated and financed the programme with partner support and pressed for SLMTA activities to be included in provincial and hospital annual plans and budgets. Decentralising programme management to the provincial level has enabled them to increase programme coverage and lower the costs.^33^

### Rwanda – Data-driven advocacy

As with many other countries, Rwanda’s laboratories suffered from chronic service disruptions as a result of reagent stock-out and equipment breakdowns from lack of maintenance. An improvement project was assigned to the SLMTA-enrolled laboratories, which tracked the number of tests not performed because of stock-out and equipment breakdowns over a three-month period. They then calculated the funds required to purchase needed reagents and maintain equipment, along with the revenue that would have been generated from these tests, finding that the missed income was far greater than the cost of preventing stock-out and equipment breakdowns. This return on investment analysis persuaded hospital management to prioritise reagent supplies and to contract with manufacturers to provide regular maintenance services for the laboratory equipment.^37^

### Cameroon – Expanding quality past the laboratory

In Cameroon, management at one hospital witnessed the transformation of its laboratory after SLMTA and undertook to extend the quality into other units of the hospital. They formed their own quality improvement teams, which have reported improved hospital cleanliness, reduced patient waiting times, greater patient satisfaction, development of new treatment protocols and increased recognition of the importance of patient safety. Additionally, a reduction in infection rates and stillbirths, as well as an increase in the number of patients served and hospital revenue, have been observed.^38^

### Zimbabwe – Overcoming contextual challenges

Zimbabwe has suffered economic crises in the past few decades, resulting in deterioration of the healthcare system and a shortage of human resources. Participants in its two SLMTA cohorts have identified creative solutions to overcome the extensive logistic and resource challenges. For example, standard operating procedures were hand-written in exercise books, Levy-Jennings charts were plotted manually and a paper-based system was used where computerised Laboratory Information Systems were not available. Hospitals recognised the value of accreditation and prioritised budgets for equipment calibration, service contracts and staff vaccinations. Funding from the US President’s Emergency Plan for AIDS Relief (PEPFAR) supported the establishment of a training and mentorship department at the Zimbabwe National Quality Assurance Program Trust in order to develop local capacity to support SLMTA programme rollout and continued quality improvement for laboratory services.^39^

## SLMTA’s global reach and influence outside Africa

The SLMTA-driven laboratory quality improvement achieved in Africa has inspired countries in other regions to follow suit, even in the absence of a regional or national accreditation preparedness scheme such as WHO AFRO’s SLIPTA. Outside the continent of Africa, 24 countries from the Caribbean Region, Central and South America and Southeast Asia have adopted the SLMTA programme and have used the SLIPTA checklist to measure gaps and the progress of enrolled laboratories. The Caribbean Region, comprising many island countries with diverse geography, people, size and economy, has implemented SLMTA in 12 countries.^25^ After completing the SLMTA programme, Bahama’s National HIV Reference Laboratory was accredited and two other enrolled laboratories in the region are also seeking international accreditation.^40^ In Southeast Asia, impressive results have also been observed in Cambodia and Vietnam, where one provincial laboratory that tests clinical as well as food and environmental samples was accredited to ISO 17025 in 2013.^25^ A desire to automate data collection, analyse and manage SLIPTA audit data more efficiently and to enable real-time graphical display of actionable results at audited facilities led to the development of a multi-lingual electronic tool in Vietnam.^41^ This tool has been shared with the global SLMTA community. In Latin America, a partnership was forged where 14 military laboratories from eight countries in the region were enrolled in PROMELA (*Programa de Mejoramiento de Laboratorios de las Fuerzas Armadas de Latinoamérica*), an overarching laboratory improvement programme using SLMTA as its principle training tool in addition to other practical laboratory training and biosafety and/or infection control training. The fact that two Africa-based master trainers (one Anglophone, one Lusophone) came to assist in the first Spanish-speaking training-of-trainers in Latin America underscores the benefits of standardised training and highlights SLMTA’s true global nature and its far-reaching network across borders and continents.

## Lessons learned

Throughout the SLMTA rollout, countries have overcome many challenges such as attrition of SLMTA-trained staff, encouraging the entire laboratory to work as a team, engaging hospital management, and insufficient mentorship capacity. Table 2 summarises the most common challenges and offers corresponding recommendations to help guide future implementation. Despite the challenges, SLMTA has worked successfully by demonstrating that with resolve, commitment and ingenuity, laboratory teams in developing countries can improve their service delivery using existing limited resources. It also demonstrates that starting with small tangible improvements (‘low-hanging fruit’) and gradually building upon early successes can boost laboratory teams’ confidence and motivate them to tackle the harder issues. This strategy is similar to the ‘Little Steps’ approach^42^ that has been shown to be effective in sustaining healthcare quality improvement efforts in developing countries.

**TABLE 2:** Common challenges and recommendations for SLMTA implementation.

Common challenges	Recommendations
Number of labs enrolled in each cohort of SLMTA: What is the best way to achieve nation-wide impact whilst ensuring each laboratory receives sufficient support and attention?	Limit the number of laboratories according to available financial, logistical, and human resources. Use the initial SLMTA-enrolled laboratories to identify problems most likely to affect other laboratories in the country. Present recommendations to upper management and advocate for system-wide reform. Target fewer laboratories or select specific units of large laboratories. Focus on strengthening those laboratories or units to become centres of excellence and twin them with other laboratories or units.
**Programme disruptions:** How can delays and disruptions during SLMTA implementation be minimised?	Before implementation, identify costs of the entire process, including all activities necessary to achieve accreditation preparedness. Budget resources accordingly. Define and agree on roles and responsibilities with all parties involved. Set dates of all programme activities during planning and adhere to the schedules. Request authorisation for budget, travel dates, release of trainers at the beginning of the programme.
**High staff turnover:** How can staff turnover be minimised during the SLMTA process?	The Ministry of Health and hospital management should be enlisted to help reduce reassignment during SLMTA implementation. Consider signing a Memorandum of Understanding with heads of the participating institutions to confirm commitment. Sites should not be enrolled if management does not agree to keep staff in current positions for the duration of the programme. Minimise the impact of turnover by training more than one person from each site.
**Non-SLMTA staff involvement:** How can staff members not involved in the SLMTA training be engaged for the overall improvement effort?	Require those who attend the SLMTA workshops to share their knowledge and tools with their colleagues when they return home. Hospital and laboratory management must be engaged and mandate that improvement projects involve all laboratory staff. Treat all the laboratory staff as a team; acknowledge, motivate, and encourage them for their effort and progress.
**Hospital management:** What is the best way to engage hospital management?	Identify a clinician who is a champion for the laboratory, and enroll that person in SLMTA. Communicate with the hospital administration, keeping them informed on issues and progress. Publicize the laboratory’s success stories. Conduct the SLMTA activity “Meet the Clinicians” on site to facilitate communication between laboratory staff and clinicians.
**Site support and mentoring:** What is the best way to ensure that each laboratory receives sufficient mentorship support, given limited mentoring capacity and resources?	Limit the number of laboratories enrolled based on the available resources required for on-site support and mentoring. Establish a structured mentorship programme using local mentors who have been carefully selected and trained. Clearly define, measure, and report outcomes of mentorship engagement.
**Program sustainability:** How can SLMTA become self-sustaining within a country?	Establish or strengthen quality management systems coordination within the existing Ministry of Health structure. Decentralise programme management to provincial levels to increase programme coverage whilst lowering cost. Integrate SLMTA into pre-service curriculum for laboratory professionals. Select and train laboratory managers or other qualified individuals as mentors within their own laboratories. Conduct in-country training-of-trainers to develop a cadre of local SLMTA implementers for continuous implementation. Reduce programme costs by using health facilities for training, rather than renting meeting space. Integrate small ‘bite-size’ training sessions into established laboratory routines, such as teaching one activity during weekly staff meetings.

SLMTA, Strengthening Laboratory Management Toward Accreditation.

Within a few years, SLMTA has demonstrated its transformative power, emerging as a flagship programme for laboratory system strengthening in PEPFAR-supported countries. A recent 2013 Institute of Medicine report^43^ recognised that improvement of laboratories under PEPFAR support and guidance has been a signature achievement. In addition, it states that:

PEPFAR’s laboratory efforts have had a fundamental and substantial impact on laboratory capacity in countries. This laboratory infrastructure has been, and continues to be, leveraged to improve the functioning of countries’ entire health systems.^43^

As laboratories do not exist in a vacuum, there have been calls^38,44^ for the SLMTA model to be adapted for the clinical settings in developing countries, with a goal toward overall hospital accreditation. This will ensure the sustainability of laboratory improvements and accreditation, and boost the centrality of quality management systems in hospital facilities, resulting in better patient care.

SLMTA implementation has been supported primarily with PEPFAR resources. To ensure its longevity and viability beyond PEPFAR, countries must work hard to integrate the SLMTA components into normal laboratory operations, decentralise programme planning and budgeting to the provincial or lower level, look for ways to be financially self-sufficient (such as charging enrollment fees for privately-owned laboratories) and incorporate the curriculum into pre-service education.

## Conclusion

After five years of implementation, SLMTA has proven to be an effective programme for the strengthening of laboratory health systems, with a focus on building management capacity in order to achieve quality services for improved patient care. Evidence to date has indicated widespread success of the programme in its ability to facilitate continuous quality improvement in the enrolled laboratories. SLMTA has the unique potential to help laboratories make progress through the SLIPTA process, improve quality of services and subsequently achieve accreditation to ISO 15189.
